# Aerobic oxidation of methane significantly reduces global diffusive methane emissions from shallow marine waters

**DOI:** 10.1038/s41467-022-35082-y

**Published:** 2022-11-27

**Authors:** Shi-Hai Mao, Hong-Hai Zhang, Guang-Chao Zhuang, Xiao-Jun Li, Qiao Liu, Zhen Zhou, Wei-Lei Wang, Chun-Yang Li, Ke-Yu Lu, Xi-Ting Liu, Andrew Montgomery, Samantha B. Joye, Yu-Zhong Zhang, Gui-Peng Yang

**Affiliations:** 1 Frontiers Science Center for Deep Ocean Multispheres and Earth System, and Key Laboratory of Marine Chemistry Theory and Technology, Ministry of Education, Ocean University of China, Qingdao, 266100 China; 2 Laboratory for Marine Ecology and Environmental Science, Qingdao National Laboratory for Marine Science and Technology, Qingdao, 266237 China; 3 College of Chemistry and Chemical Engineering, Ocean University of China, Qingdao, 266100 China; 4 State Key Laboratory of Marine Environmental Science, College of Ocean and Earth Sciences, Xiamen University, Xiamen, 361102 China; 5 College of Marine Life Sciences, and Frontiers Science Center for Deep Ocean Multispheres and Earth System, Ocean University of China, Qingdao, 266100 China; 6 Department of Earth Sciences, University College London, London, WC1E 6BS UK; 7 College of Marine Geosciences, Ocean University of China, Qingdao, 266100 China; 8 Department of Chemistry and Biochemistry, Montana State University, Bozeman, MT 59717 USA; 9 Department of Marine Sciences, University of Georgia, Athens, GA 30602 USA; 10 Marine Biotechnology Research Center, State Key Laboratory of Microbial Technology, Shandong University, Qingdao, 266237 China

**Keywords:** Marine chemistry, Biogeochemistry

## Abstract

Methane is supersaturated in surface seawater and shallow coastal waters dominate global ocean methane emissions to the atmosphere. Aerobic methane oxidation (MOx) can reduce atmospheric evasion, but the magnitude and control of MOx remain poorly understood. Here we investigate methane sources and fates in the East China Sea and map global MOx rates in shallow waters by training machine-learning models. We show methane is produced during methylphosphonate decomposition under phosphate-limiting conditions and sedimentary release is also source of methane. High MOx rates observed in these productive coastal waters are correlated with methanotrophic activity and biomass. By merging the measured MOx rates with methane concentrations and other variables from a global database, we predict MOx rates and estimate that half of methane, amounting to 1.8 ± 2.7 Tg, is consumed annually in near-shore waters (<50 m), suggesting that aerobic methanotrophy is an important sink that significantly constrains global methane emissions.

## Abstract (web summary)

Aerobic oxidation is a biological sink of methane that can reduce oceanic emissions to the atmosphere. This study estimates that half of methane from total loss, amounting to 1.8 ± 2.7 Tg, is oxidized annually in global 0–50 m near-shore waters

## Introduction

Methane (CH_4_), a powerful greenhouse gas with a global warming potential > 27 times greater than that of carbon dioxide over 100 years^1^, plays a crucial role in the global carbon cycle^2^. Emissions of methane from the ocean to the atmosphere are estimated to be 6–12 Tg yr^−1^ ^3^, accounting for 1–10% of natural emissions^4^. Large amounts of methane are stored beneath the seafloor, and most of this methane is consumed by anaerobic oxidation of methane, a well-documented process that regulates methane escape from diffusion-dominated sediments^5^. In the water column, aerobic methane production from bacterial cleavage of methylated compounds, such as methylphosphonate (MPn), methylamine (MA), and dimethylsulfoniopropionate (DMSP), contributes to methane supersaturation in the surface mixed layer^6–10^. Similar to anaerobic oxidation of methane, aerobic methane oxidation (MOx) serves as a biological sink that mitigates methane emission from marine waters to the atmosphere^5,11^. However, the magnitude and control of this process remains largely unconstrained, limiting our quantitative understanding of the role of MOx in marine methane cycling. Despite representing only a minor fraction of the total ocean areas, shallow coastal waters (i.e., <50 m) dominate global oceanic methane emissions^3,12^. Dissolved methane concentrations have been documented intensively^7,12–15^, but MOx rate measurements are limited, which makes it difficult to assess the importance of biological removal in moderating atmospheric methane emission accurately.

To document the sources and fate of methane in shallow marine waters, we investigate methane fluxes, production pathways, and methanotrophic activity using a suite of biogeochemical approaches in the near-shore and outer shelf waters of the East China Sea (ECS). Furthermore, combining our MOx rates with previously published data (a total of 427 data points) in diffusion-driven coastal waters, we generate predictions of global MOx rates in shallow waters based on methane concentrations and other variables using a random regression forest (RRF) machine-learning method. We estimate that 1.8 ± 2.7 Tg methane are consumed annually by MOx in near-shore waters (<50 m). The results suggest that half of methane present in nutrient-rich shallow waters is oxidized, reducing methane emissions to the atmosphere substantially.

## Results and discussion

### Methane distribution, saturation, and flux in the East China Sea

**Fig. 1:**
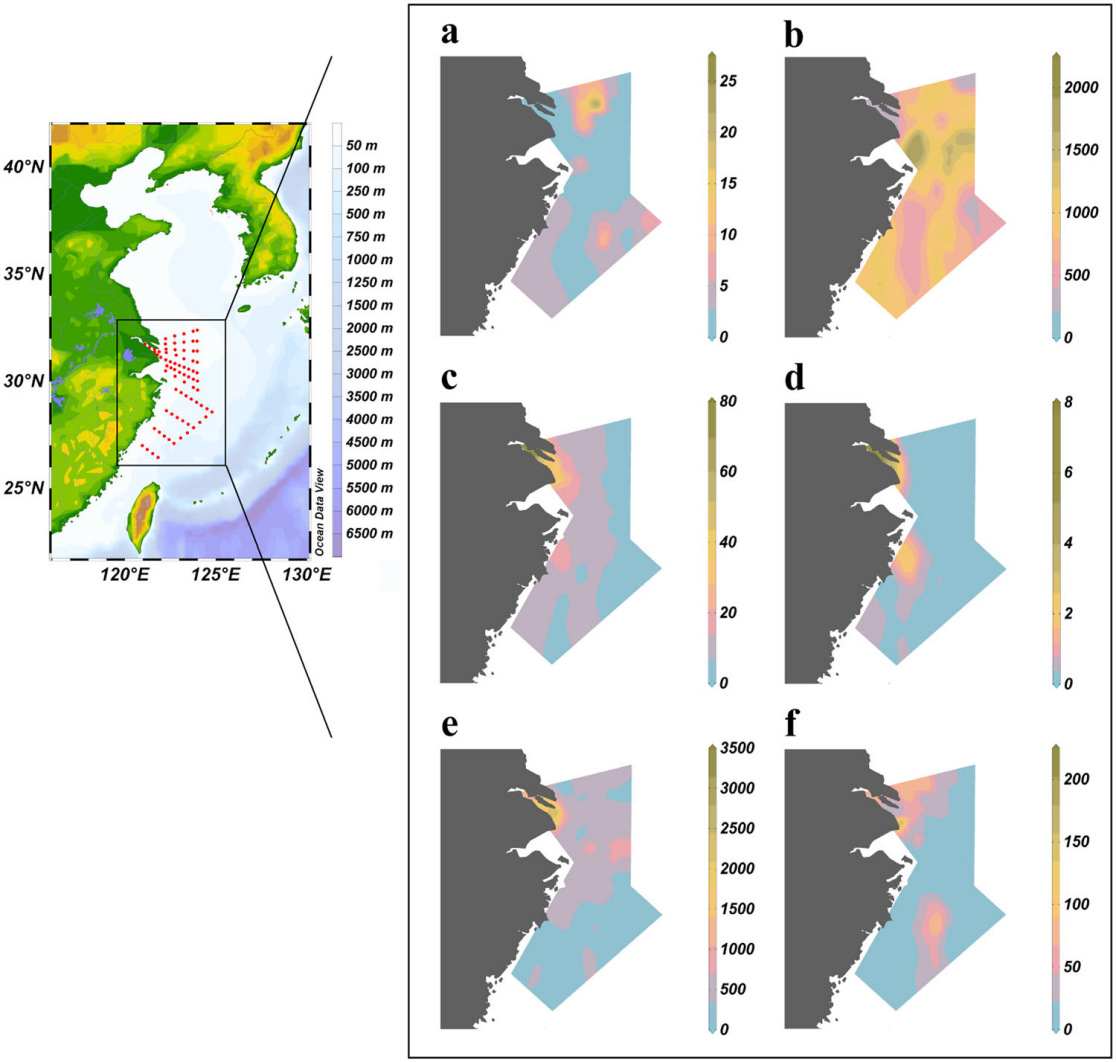
Methane concentrations, consumption rates and fluxes in the East China sea (ECS) and Yangtze River Estuary. Spatial distribution of **a** chlorophyll-*a* concentrations (Chl-*a*, μg L^−1^), **b** bacterial production rates (BP, nmol C L^−1^ d^−1^), **c** methane concentrations (CH_4_, nmol L^−1^), **d** methane oxidation rates (MOx, nmol L^−1^ d^−1^), **e** methane saturation (R, %), and **f** sea-to-air fluxes (F, μmol L^−1^ d^−1^) in surface seawater. Figure was created using Ocean Data View (version 5.5.2)^67^.

Sea-surface waters were sampled from 76 sites during two research cruises to the Yangtze river estuary and ECS (Supplementary Fig. 1). The study areas, including estuary, coast and continental shelf waters, were largely impacted by complex hydrology, physical dynamics and anthropogenic processes. High primary productivity and heterotrophic activity were observed in these areas, reflected in elevated Chl-*a* concentrations (0.6–14.5 µg L^–1^) (Fig. 1a) and high bacterial production rates (222.7–2078 nmol C L^–1^ d^–1^) (Fig. 1b). Due to significant riverine inputs, surface water nutrient concentrations at coastal sites exceeded those at the offshore sites (Supplementary Fig. 2). Given the hydrological and biogeochemical features, the Yangtze estuary and ECS represent an excellent case study for estuary-shelf continuum systems across the globe.

Methane concentrations decreased progressively from the estuary and coastal sites to the offshore waters. The concentrations were generally higher than 20 nM at the estuarine sites (maximum of 79 nM), but lower than 8 nM in waters deeper than 50 m (Fig. 1c). Methane was supersaturated with respect to atmospheric equilibrium in the surface waters at all study sites, and the saturation state ranged from 145 to 3229% (Fig. 1e). Sea-to-air fluxes varied between 2.7 and 99.6 µmol m^–2^ d^–1^ (average: 20.6 ± 22.3 µmol m^–2^ d^–2^) (Fig. 1f). Although the ECS represents only 0.23% of the global ocean (~7.7 × 10^5^ km^2^)^16^, the annual release of methane from the ECS (~0.09 Tg CH_4_ yr^−1^) could account for 1.4–4.1% of the global diffusive ocean-to-atmosphere methane flux (2.2–6.3 Tg yr^−1^)^3^, underscoring the disproportionate contribution of near-shore and shelf areas to global ocean methane emissions.

### Methane sources and production pathways

Methane concentrations at freshwater riverine sites (i.e., B1–B3, C1–C4; average methane concentration ~49 nM) were much higher than coastal sites, suggesting that riverine inputs could be an important source of methane in estuarine areas, a scenario frequently observed in global river estuary systems^12,15,17–19^. However, methane concentrations did not correlate well with salinity when salinity exceeded 20 (CH_4_ vs. Salinity > 20; Supplementary Fig. 3), if we excluded the sites close to the river mouth during the Yangtze River expedition (Sites A, B, C series). During the ECS expedition (Sites S series), the study sites were not significantly influenced by riverine input, as reflected from the narrow range of salinity (i.e., 29–34; Supplementary Fig. 3). Similarly, no significant relationship was observed between methane and salinity in the ECS (Sites S series; Supplementary Fig. 3). Furthermore, the average methane concentration in the Yangtze River estuary and ECS excluding the freshwater sites (~9 nM), was within the typical range in global coastal waters (0.7–20 nM)^3^. Collectively, while riverine input could contribute to the high methane observed in the river mouth, it is likely that other sources such as in situ production drive methane supersaturation beyond the estuary along the ECS.

**Fig. 2:**
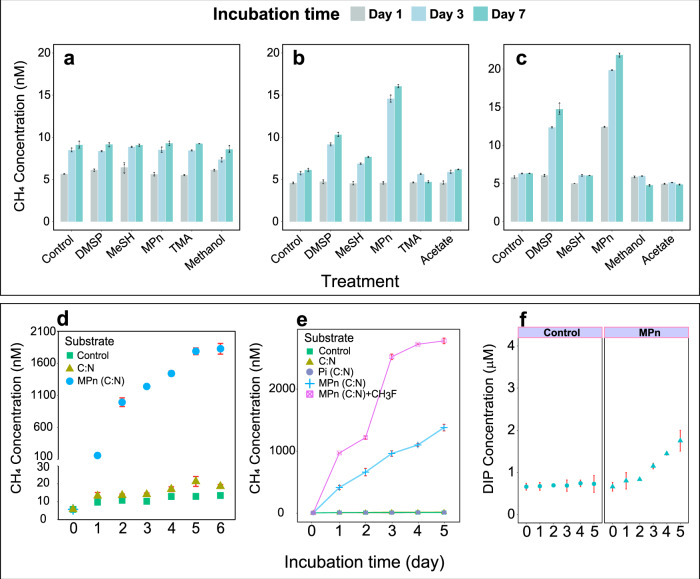
Methane production in surface seawater. CH_4_ production during incubations with additions of 1 µM acetate, 1 µM methanol, 1 µM dimethylsulfoniopropionate (DMSP), 1 µM methylphosphonate (MPn), 1 µM methanthiol (MeSH), and 1 µM trimethylamine (TMA) at sites P1 (**a**, near-shore site, water depth 19 m), P5 (**b**, offshore site, water depth 87 m) and S4 (**c**, offshore site, water depth 104 m); biological production of CH_4_ in surface waters at site S2 (near-shore site, water depth 48 m) with C:N (1060 µM glucose:160 µM nitrate) or C:N and 10 µM MPn additions (**d**); CH_4_ production in offshore waters at P5 station amended with 10 µM inorganic phosphorus (Pi), 10 µM MPn and 10 µM MPn + fluoromethane (CH_3_F) after adjusting C:N ratio (**e**); changes of dissolved inorganic phosphorus (DIP) concentrations in offshore surface waters at site S3 (offshore site, water depth 87.5 m) with 5 µM MPn addition (**f**). Data are presented as mean values and error bars represent standard deviation of triplicate samples.

To elucidate potential production pathways responsible for methane supersaturation, we conducted incubation experiments with additions of methanogenic substrates in near-shore and offshore waters. Compared to the untreated controls, methane concentrations did not increase following amendment of 1 µM acetate, methanol, methanethiol, or trimethylamine at any sites over 7-day incubations (Fig. 2a–c), indicating that anaerobic methanogenic pathways were not important in the oxic waters of the ECS. This notion is supported by metagenomic analysis since methyl coenzyme M reductase (*mcr*A), the functional gene of methanogens was not detected in our environmental samples (Supplementary Table 1). The activity of canonical methanogenesis was not captured since those processes occur only in anoxic micro-niches inside sinking particles or in zooplankton guts^20,21^. Likewise, methane concentrations did not change during incubations with 1 µM DMSP or MPn in near-shore waters (Fig. 2a). However, significant methane production was observed in offshore waters following addition of MPn and DMSP. In those treatments, final methane concentrations were 2–4 times higher than those observed in unamended controls (Fig. 2b, c). These results suggest that methane is produced under aerobic conditions through the degradation of MPn or DMSP, particularly under nutrient limitation. Indeed, the average phosphate concentration at near-shore sites was >3 times of that at offshore sites (Supplementary Fig. 2b). The addition of MPn increased dissolved inorganic phosphate concentrations in offshore waters, indicating MPn mineralization boosted the availability of inorganic phosphate (Fig. 2f).

To generate P-limiting conditions, we adjusted the C:N:P ratio by adding 1060 µM glucose and 160 µM nitrate simultaneously to near-shore water samples. Intriguingly, after 6-day incubations, methane concentrations (~20 nM) were slightly higher in C + N amended samples than unadjusted controls (~13 nM) (Fig. 2d). Addition of 10 µM MPn to the C + N treatment led to production of 1800 nM methane. In the offshore waters, methane did not accumulate with the addition of 10 µM inorganic phosphate in the C + N treatment. However, significant methane production (>1 µM) was observed in the C + N treatment following the addition of 10 µM MPn (Fig. 2e). These observations together confirmed that MPn cleavage was the dominant pathway for aerobic methane production under P-limited conditions. Relative abundances of genes encoding MPn synthesis and C-P lyase pathway were consistently elevated in ECS surface waters (Supplementary Table 1). Phosphoenolpyruvate mutase gene (*pepM*)^22^ and methylphosphonate synthase (*MPnS*)^23^, genes involved in phosphonate biosynthesis, were present in relatively high abundance. The phosphonate degrading (*phn*) gene clusters (e.g., *phnC*-*E*, *phnI*-*M*) were also detected; this gene is responsible for cleaving the C–P bond in phosphonates, releasing methane as a byproduct^24–26^.

In addition to endogenous methane production in waters, methane enters the system from sedimentary sources, as reflected in sediment core incubations. Sediment cores were collected, and the overlying water was removed and replaced with 0.2 µm filtered bottom water. Methane concentrations in the overlying water increased dramatically during the first 24 h and reached 163.3 ± 12.7 nM after a 3 day incubation (Supplementary Fig. 4). A previous study estimated the average diffusive sediment-water methane flux of 1.11 ± 0.40 µmol m^−2^ d^−1^ along the continental shelf of the ECS^17^. To quantitively estimate methane emissions from sediments, we extrapolated this reported average diffusive flux to the area of ECS. The calculation suggests that ~0.005 Tg yr^−1^ of methane enters the water column from sediments, making sediment release a source of methane to the ECS.

### Methanotrophic activity and spatial variation

Methane concentrations in marine waters reflect an imbalance between sources and sinks from interactions of multiple processes including physical transport, production and consumption. We measured MOx rates using ^3^H labeled radioactive methane tracer. The rate constant, *k*, which is a proxy for methanotrophic biomass^27^, spanned a wide range from 0.0019 to 0.1173 d^−1^, with an average of 0.034 ± 0.027 d^−1^, which lies among the high values measured previously^18,27–31^. MOx rates varied between 0.01 and 7.5 nmol L^−1^ d^−1^ (Fig. 1d), and fell within reported ranges^14,18,27–31^, and were comparable to rates observed in a number of methane seep impacted waters^32–34^. In contrast to the high rates driven by elevated methane concentrations near seeps^33,35–37^, rapid turnover times and correspondingly high MOx rates reflect the inherent capacity of methane oxidizing communities to consume methane at the low concentrations typical of the ECS. Methanotrophic activity decreased from coastal and estuarine sites to the offshore waters; *k* values and MOx rates were 3.5 and 29 times higher in estuarine waters than offshore waters (water depth > 50 m), respectively. MOx rates did not correlate with bacterial production rates, although a previous study demonstrated increasing heterotroph richness can stimulate methanotrophic activity^38^. Assuming methanotrophs incorporate about 25% of methane carbon into biomass^39^, we estimated that methane assimilation accounted for 0.03% of bacterial carbon production, indicating an insignificant contribution of methane to bacterial carbon production.

The rapid rates of methane consumption along the ECS suggest microbial consumption is an important removal pathway that moderates methane emission to the atmosphere. The addition of CH_3_F, an inhibitor of methane oxidation^40^, during methane production incubations, increased methane accumulation significantly (~2800 nM) (Fig. 2e). Comparing treatments with and without (~1400 nM CH_4_) CH_3_F showed that an average of 56% methane was oxidized concurrently during incubations.

Due to the lack of depth profiles of MOx rates in the ECS, we used surface water MOx rates to approximate the depth-integrated methane oxidation rates. We note that this calculation could represent an initial estimate, although MOx rates did not seem to vary significantly with depth in some shallow waters (Supplementary Fig. 5 and refs. 41, 42). Depth-integrated MOx rates ranged from 0.7 µmol m^−2^ d^−1^ to 93.5 µmol m^−2^ d^−1^ (average: 21.34 ± 19.41 µmol m^−2^ d^−1^), and accounted for removal of ~0.10 Tg CH_4_ yr^−1^ across the system. This approximation was generally consistent with the calculated depth-integrated MOx rates (22.08 ± 7.92 µmol m^−2^ d^−1^) using the predicted MOx rates from multiple depth profiles with the RRF model (see next section). The similar fluxes between MOx (~0.10 Tg CH_4_ yr^−1^) and sea-air exchange (~0.09 Tg CH_4_ yr^−1^), suggested that ~51% of methane was removed by MOx, signifying that microbial oxidation is an important methane sink in the ECS. This finding is in contrast to a number of previous studies, which demonstrated that MOx was not a significant sink for methane and that a large fraction of methane escaped to the atmosphere^19,28^. For example, it was found that >95% of methane could be released to the atmosphere in the hypoxic zone of the Louisiana shelf since the methanotrophic community is inefficient to remove methane before ventilation^28^.

### Predicted global MOx rates in marine waters

**Fig. 3:**
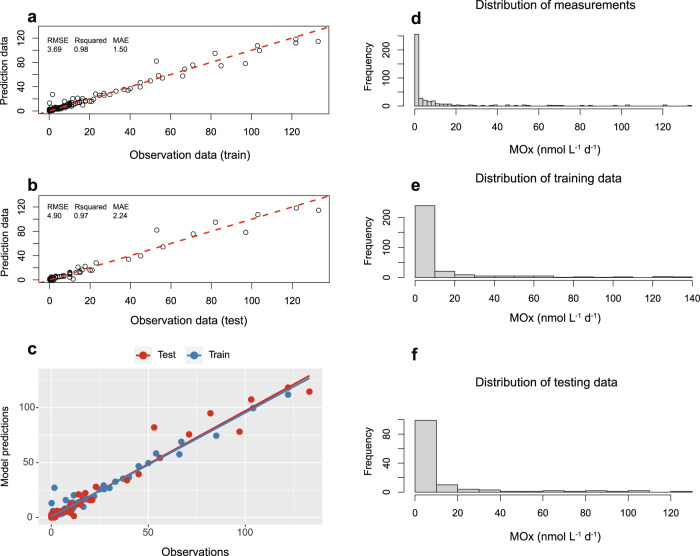
Comparison of measured methane oxidation (MOx) rates to model predictions. **a** Tracer-tracer plots of measurements and training data. We randomly draw 75% of the measurements to train our random regression forest; **b** Tracer-tracer plots of measurements and testing data (the rest 25%); **c** Centralized presentation of predicted data from training and testing data; **d** Frequency distribution of all measured MOx rates; **e** Frequency distribution of MOx rates used to train the model; **f** Frequency distribution of MOx rates used for external testing. The red dashed line is a 1:1 line. RMSE (Root Mean Squared Error), R squared (Coefficient of determination) and MAE (Mean Absolute Error) are prediction evaluation metrics for the dataset.

**Fig. 4:**
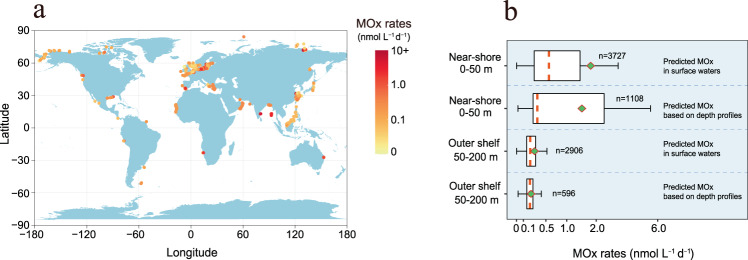
Machine-learning prediction of global methane oxidation (MOx) rates. **a** Global map of predicted MOx rates in surface water of near-shore (0–50 m) and outer shelf (50–200 m). The rates were generated by the random regression forest (RRF) method based on the published dataset containing methane, temperature, salinity, and depth (6633 datapoint) in diffusion-driven waters. **b** Probability distributions of predicted MOx rates in surface water and at depths in the near-shore (0–50 m) and outer shelf (50–200 m). MOx rates at depths were predicted using the depth profiles of methane and other variables from global database. Red dashed lines are the median values, and the green diamonds are the mean values. The lengths of the boxes represent the interquartile range, with whiskers spanning the maximum and minimum excluding outliers.

Aerobic methane oxidation plays a significant role in controlling methane emission from the ocean to the atmosphere. However, due to scarce MOx rate measurements, quantitative estimates of this process on a global scale are unconstrained. Therefore, we extrapolated global MOx rates using RRF machine-learning models. As most MOx rates were measured along continental shelves and in estuarine waters, a global database of MOx rates (*n* = 427) from diffusion-driven systems (i.e., excluding data from unique environments such as methane seeps) from this and previous studies (Supplementary Table 2) was compiled for training RRF models. Skillful prediction models were constructed by assessing the importance of predictor variables exploiting pattern similarities between MOx and other environmental factors including methane, salinity, temperature, and depth (Supplementary Fig. 6). A random subset of 75% of the dataset was used in the RRF ensemble for training, leaving 25% of the data for validating the model (*R*^2^ > 0.9) (Fig. 3). After training, the RRF model was used to generate the prediction and global map of MOx rates in surface waters (0–200 m) based on methane concentrations, temperature, salinity, and depth (6633 individual grid data points; Fig. 4a) retrieved from the database. The predicted MOx rates in surface water ranged between 0.002 and 13.88 nmol L^−1^ d^−1^ (average: 1.78 ± 2.74 nmol L^−1^ d^−1^) in near-shore environments (0–50 m) and between 0.001 and 2.33 nmol L^−1^ d^−1^ (average: 0.28 ± 0.26 nmol L^−1^ d^−1^) in outer shelf regions (50–200 m) (Fig. 4b). Like the methane distribution, MOx rates increased sharply towards coastlines. The predicted high MOx rates in near-shore environments suggest that this process could serve as an important biological sink to reduce the global diffusive methane emissions from shallow waters. Furthermore, we also predicted depth profiles of MOx rates in the ECS using this model based on the previous measurements of methane concentrations and other parameters in the ECS^43^ (Supplementary Table 3). The predicted depth-integrated MOx rates (22.08 ± 7.92 µmol m^−2^ d^−1^) were similar to the measured values, verifying the validity of our model. Modeled methane consumption rates (~0.10 Tg CH_4_ yr^−1^) again confirmed the significance of MOx as a methane sink in the ECS.

### Global methane oxidation budget and importance in shallow waters

The global ocean represents a highly uncertain source of the atmospheric methane and total oceanic methane emissions were estimated to be 6–12 Tg CH_4_ yr^−1^ ^3^. Despite accounting for only ~3% of the ocean by area, near-shore environments (0–50 m) contribute the largest diffusive methane flux, and the emissions from these areas have been estimated to be between 0.8 and 3.8 Tg yr^−1^ (2.0 ± 1.45 Tg yr^−1^)^3^. To calculate the depth-integrated MOx rates in the water column of shallow waters (0–50 m), we also modeled the MOx rates at different depths using vertical profiles of methane and other variables from the database (1108 data points). The predicted MOx rates at depths ranged between 0.02 and 9.02 nmol L^−1^ d^−1^ (average: 1.46 ± 1.84 nmol L^−1^ d^−1^) (Fig. 4b). Together with the surface MOx rates, we obtained an average of 1.71 ± 2.57 nmol L^−1^ d^−1^ from total modeled MOx rates throughout the 0–50 m water columns (Table 1). Combining an average depth of 0–50 m computed from the ETOPO2 high-resolution bathymetry data (~16.5 m) and the total area of 0–50 m waters (1.09 × 10^7^ km^2^, assuming 3% of the ocean area^3,12^), we estimated that methane consumed through microbial oxidation in near-shore environments sums to 1.80 ± 2.70 Tg yr^−1^ (10–90th percentile range: 0.11–6.37 Tg yr^−1^) (Table 1). These values were similar to reported diffusive methane emission fluxes (i.e., 2.0 ± 1.45 Tg yr^−1^)^3^, suggesting nearly half of methane is oxidized prior to atmospheric ventilation. Contrary to previous observations in coastal systems, where MOx appears inadequate in controlling methane flux^19,28,44^, these findings also challenge our current understanding and highlight the importance of MOx in methane cycling in shallow near-shore waters. Although the relative importance of ventilation versus MOx may vary depending on physical mixing or meteorological conditions at an individual site, on a global scale, the total amount of methane consumed by MOx in 0–50 m waters, accounts for 47% of the total loss (sum of MOx and sea-air flux) in near-shore environments based on our calculations. This value is similar to our observations in ECS (51% methane oxidized before ventilation), suggesting that microbial oxidation of methane provides an efficient internal methane sink that limits the diffusive flux from global shallow waters to the atmosphere.

**Table 1:** Estimation of methane consumed through aerobic methane oxidation (MOx) in global 0–50 m near-shore waters

Near-shore waters (0–50 m)	Average MOx Rate	Average water depth^a^	Methane consumed by MOx		Diffusive flux^d^	MOx/Total loss^e^
	(nmol L^−1^ d^−1^)	(m)	(Tg yr^−1^)		(Tg yr^−1^)	(%)
Surface water (*n* = 3727)	1.78 ± 2.74	16.5	1.87 ± 2.88^b^	4.16 ± 6.40^c^	2.00 ± 1.45	48.3
Depth profiles (*n* = 1108)	1.46 ± 1.84	16.5	1.53 ± 1.93^b^	3.41 ± 4.30^c^	2.00 ± 1.45	43.3
Surface water + Depth profiles (*n* = 4835)	1.71 ± 2.57	16.5	1.80 ± 2.70^b^	4.00 ± 6.00^c^	2.00 ± 1.45	47.4

^a^The average depth of 0–50 m was computed from the ETOPO2 high-resolution bathymetry (see the “Methods” section).

^b^Methane consumed by MOx was calculated based on the area of near-shore waters accounting for 3% of the ocean area (i.e., ~1.09 × 10^7^ km^2^)^3^.

^c^Methane consumed by MOx was calculated based on the area of near-shore waters derived from the seafloor geomorphic features map of the global ocean (i.e., ~2.4 × 10^7^ km^2^)^66^.

^d^Diffusive fluxes was reported based on the area of near-shore waters accounting for 3% of the ocean area^3^.

^e^MOx/Total loss = Methane consumed by MOx^b^/(Methane consumed by MOx^b^ + diffusive fluxes^d^).

Our work suggests that shallow shelf waters dynamically cycle methane with multiple sources and rapid removal throughout global oceans. Coastal ecosystems are more prone to anthropogenic perturbations and they are characterized by high productivity, elevated microbial activity, and altered nutrient cycling due to human alterations of nutrient loading and changes in oceanographic conditions. In the ECS, methane was highly oversaturated in surface waters and incubation experiments in tandem with molecular evidence confirmed that methane production from MPn metabolism under P limitation is a source of endogenous methane.

High sedimentation rates and rapid accumulation of labile organic matter can alter mineralization pathways and geochemical zonation, resulting in increased production and diffusive flux of methane in surface sediments. Once produced, rapid MOx and sea-air exchange efficiently remove methane from the water column and maintain the dynamic balance of methane in the upper ocean. MOx accounted for 51% of total methane loss (the sum of MOx and sea-air flux) in ECS waters. Globally, high methanotrophic activity in near-shore waters can consume 1.80 ± 2.70 Tg yr^−1^ methane, compared to 2.0 ± 1.45 Tg yr^−1^ that is ventilated to the atmosphere^3^, suggesting that methanotrophy is a significant methane sink that consumes approximately half of methane from total loss in near-shore coastal waters. This first approximation of shallow methane oxidation and its role in the global methane budget reshapes our view of methane cycling in shallow methane-rich coastal waters.

## Methods

### Study area and sample collection

Seawater samples were collected from a total of 76 sites in the Yangtze River Estuary and East China Sea (ECS) onboard the Research Vessels (R/Vs) “Runjiang 1”, “Zheyuke 2” and “Xiangyanghong 18” during expeditions in March-April and July 2021 (Supplementary Fig. 1). The ECS is one of the most productive marginal seas in the West Pacific, with an average depth of 349 m. Significant input from the Yangtze River and complex hydrological conditions influence nutrient levels and marine productivity in the ECS, making the ECS highly dynamic among global shelf regions^45,46^. A Seabird 911 CTD-Niskin rosette system was used for water collection, and individual samples were collected for the determination of methane and nutrient concentrations, rates of aerobic methane oxidation, bacterial production, metagenomics, and incubation experiments.

### Methane concentrations and geochemical analyses

Seawater methane concentrations were measured using a cryogenic purge-and-trap system connected to an Agilent GC-8890 gas chromatography with a flame ionization detector^15^. Briefly, 50 mL seawater was introduced into a glass chamber and purged with high-pure N_2_ at a flow rate of 80 mL min^−1^ for 5 min. The extracted gas was trapped in a U-shape stainless steel trap packed with Porapak Q (80/100 mesh) and immersed in liquid nitrogen. After purging, the Porapak Q trap was transferred to a boiling water bath and trapped methane gas was released into the GC. Methane samples during incubation experiments were analyzed using the headspace method, followed by gas chromatography^47^. Calibration was conducted with different concentrations of certified methane standards (National Institute of Metrology, China). Nutrient concentrations were determined with spectrophotometric methods using an autoanalyzer (SEAL AA3)^48^.

### Incubation experiment

To identify potential methane production pathways in oxic seawater, incubation experiments were conducted at different sites (Supplementary Table 4) in 125 mL acid-washed and sterilized serum vials filled with seawater; 40 mL headspace was created with high purity helium (99.999%). Different precursor substrates including MPn, trimethylamine, DMSP, methanol, methanethiol, and acetate were added to the vials to a final concentration of 1 µM (Supplementary Table 5). Samples were incubated for 1, 3 and 7 days (d) at in situ temperature and headspace methane concentrations were measured with a GC as described above. In another set of experiments (Supplementary Table 5), C:N:P ratios were adjusted by adding glucose (final concentration: 1060 µM) and nitrate (final concentration: 160 µM) with/without MPn or inorganic phosphate (Pi) (10 µM) to further assess the utilization of MPn under nutrient limiting conditions. Methyl fluoride (CH_3_F) was added to the headspace (1 kPa) in the C + N + MPn treatment to inhibit methane oxidation during incubation^40^. Methane concentrations were measured at different time points as described above.

To identify methane source from the sediment, sediment samples were collected using a box core sampler onboard the R/V “Runjiang 1” from a Yangtze River Estuary site (longitude: 122.74° E, latitude: 31.79° N; water depth: 34 m) and duplicated ~30 cm sediment cores were taken using a plexiglass tube (~7 cm i.d.). After collection, overlying water was carefully removed without disturbing the surficial sediments and 1200 mL 0.2 µm filtered bottom water from the same site was introduced. The cores were subsequently sealed with thick rubber stoppers fitted with two sampling valves. At each time point (1, 2, and 3 days), 50 mL of overlying water was sampled (*n* = 2) using a syringe through the valves and methane concentrations were measured in these subsamples.

### Methane saturation and sea-air flux calculations

The saturation of dissolved methane (*R*, %) was calculated using the methane concentrations measured in the surface water divided by the equilibrium concentrations with the atmospheric methane, which were obtained from the NOAA / ESRL Global Observations Project^49^ and corrected for in situ temperature and salinity^47^.

Sea-to-air methane flux (µmol m^−2^ d^−1^) was calculated using the W2014 model with in situ wind speed from a shipboard meteorological instrument (RM Young, Traverse City, MI) at 10 m above the sea surface, methane concentration and Schmidt number, the details of which were described in ref. 50.

### Aerobic methane oxidation rate measurements with ^3^H labeled tracers

We used a radiotracer approach to determine the water column MOx rate by adding ^3^H labeled methane to seawater samples and quantifying the conversion of ^3^H–CH_4_ to ^3^H–H_2_O^27^. Four replicates of seawater samples (triplicate live samples and one killed control) were filled into 15 mL Hungate tubes without headspace. A 100-µL aliquot of ^3^H–CH_4_ gas (~0.7 TBq mmoL^−1^) was injected through a stopper into each replicate by displacing the same volume of seawater. Controls were killed with a final concentration of 3.7% formaldehyde before tracer additions. Samples were incubated in the dark at in situ temperature for 24 or 48 h. After incubation, the total radioactivity (^3^H–CH_4_ and ^3^H–H_2_O) added to the sample and the product of the oxidation (^3^H–H_2_O) were measured separately in each control and sample. To determine the total radioactivity, a ~100 μL subsample was pipetted from each rate tube to a 2 mL plastic scintillation vial containing ~1.7 mL scintillation cocktail. The sample was counted using a Tri-Carb 3110TR liquid scintillation counter and the total added radioactivity (DPM-^3^H-CH_4_ + DPM-^3^H-H_2_O) was calculated (~20 kBq). The remaining sample was transferred to a 45-mL falcon tube containing 2 mL 37% formaldehyde and purged with N_2_ for 45 min to remove the unused ^3^H–CH_4_. After that, an aliquot of each sample was taken, and the produced ^3^H_2_O was quantified after addition of scintillation cocktail using the Tri-Carb 3110TR scintillation counter. The methane turnover rate constant (*k*, d^−1^) was calculated as the ratio of the recovered ^3^H_2_O divided by total amount of ^3^H–CH_4_ added versus the incubation time. MOx rates (nmol L^−1^ d^−1^) were obtained by multiplying *k* values by the in situ methane concentration. Heterotrophic bacterial production (BP) rates were determined by quantifying the incorporation of ^3^H-leucine into microbial protein synthesis^51,52^, the details of which were described previously^53^. Bacterial carbon production (BCP) rates were calculated based on the theoretical leucine to carbon conversion factor^51,54^. The contributions of methane incorporation to BCP were estimated using the equation of Con_*BCP*_ = 0.25× MOx/BCP, assuming methanotrophs incorporate about 25% of methane carbon into biomass^39^.

### Machine-learning prediction of global MOx rates

A global database of MOx rate data measured from diffusion-driven waters in the estuaries and continental shelves (excluding data from seeps; *n* = 427) were compiled for training machine-learning models (Supplementary Table 2). As no consistent linear relationships were observed between MOx and methane concentration (Supplementary Figs. 6 and 7), other variables such as salinity and temperature were required for training and MOx rate data without them were rejected. The collected database was used to train random regression forest models (RRF) based on CART algorithm. Skillful prediction models for MOx were constructed by exploiting pattern similarities between MOx and other parameters including methane, salinity, temperature, and depth after assessing the importance of predictor variables (Supplementary Figs. 6 and 8). A random subset of 75% of the dataset was used in the RRF ensemble for training, leaving 25% of the data for validating the model (*R*^2^ > 0.9). The RRF model was set up with 105 regression trees, and the individual regression tree was constructed with a maximum of 167 decision forks to maximize the predictive power of the model (Supplementary Fig. 6c, d). Data from different areas were used separately as training or validation data to examine the fit, to rule out the possibility that random sample data of training and validation from the same areas led to a high fit of the prediction model. All RRF ensembles reproduced the validation data for different areas with *R* > 0.8. After training, RRF model was used to generate the prediction of MOx rates based on methane concentrations, temperature, salinity and depth. The global distribution of predicted MOx rates in surface waters was mapped, which contains 6633 grid data points from the global database (excluding seeps) in the near-shore waters (0–50 m) and outer shelf (50–200 m) (Fig. 4a). For the rates predicted from monitoring stations with long time series or same stations at different investigation times, the average rates were calculated and mapped for this site.

Since seeps are mostly located with water depth >50 m, MOx rates were not impacted by seeps in shallow waters (0–50 m). To exclude seeps for waters depths between 50–200 m, data were filtered based on the methane concentrations: seep ≥10 nM CH_4_ and non-seep <10 nM CH_4_, as methane concentrations were typically <10 nM in non-seep areas based on this study and a number of previous observations^15,27,55,56^. The average MOx rates from different depths throughout water column in the near-shore and out-shelf waters were also predicted using vertical profiles of methane and other variables from the database (Fig. 4b).

The dataset of methane concentrations used for the RFF model predictions was retrieved mainly from the MarinE MethanE and NiTrous Oxide (MEMENTO) database. We downloaded all measured dissolved methane concentration data during the time interval September 1976 to December 2016 from this database, and references of each data contribution have been recorded at MEMENTO^57^. Data from other publications were also included in the database to expand data coverage and generate more reliable global predictions^6,58,59^. Due to the importance of physical variables for MOx rate prediction, only data points with accompanying methane concentration, temperature, salinity, and depth data were retained. To calculate the depth integrated MOx rates in global near-shore waters, we downloaded global seafloor depth data (*n* = 58,330,800) from the ETOPO2 high-resolution bathymetry from the US National Geophysical Data Center (NGDC)^60^ (resolution: 0.033°). Sites with depths ≤ 50 m were selected to determine the mean seafloor depth (*D*_shallow_) in the near-shore areas using the equation of ${D}_{{{{{{\rm{shallow}}}}}}}=\frac{1}{n}\mathop{\sum }\limits_{i=1}^{n}f(i)$, where *f* (i) is the site depth and n is the number of near-shore sites based on 0.033° resolution (n = 421473) filtered by the dplyr R software package.

### Bioinformatic analyses of genes involved in methane metabolism

Total genomic DNA was extracted from samples using the E.Z.N.A.® Soil/Water DNA Kit (Omega Bio-tek, Norcross, GA, USA) according to manufacturer’s instructions. Metagenomic shotgun sequencing libraries were constructed and sequenced at Wefind Biotechnology Co., Ltd (Wuhan, China). The raw reads from metagenome sequencing were used to generate clean reads by removing adapter sequences, trimming and removing low-quality reads (reads with N bases, a minimum length threshold of 50 bp and a minimum quality threshold of 20) using the fastp^61^ on the free online platform of Majorbio Cloud Platform^62^. These high-quality reads were then assembled to contigs using MEGAHIT^63^.

The functionally ratified protein sequences, namely McrA, MmoX, MmoY, MpnS, PepM, PhnA, PhnB, PhnC, PhnD, PhnE, PhnF, PhnG, PhnH, PhnI, PhnJ, PhnK, PhnL, PhnM, PhnN, PhnO, Poly (ethylene terephthalate) hydrolase and Mono (2-hydroxyethyl) terephthalate hydrolase were obtained from the National Center for Biotechnology Information (NCBI) database^64^ (Supplementary Dataset 1). Homologs of the proteins in metagenomes were obtained using BLASTP with cutoff values of identity >30% and e-value <1e-50. The relative abundance of these proteins was normalized as described previously^65^. For taxonomic profiling, the amino acid sequences of predicted methane metabolism related genes from these metagenomes were extracted using scripts compiled in Python code and aligned against the non-redundant protein sequences (nr) database using BLASTP. The best hit of each query sequence was retrieved, and its taxon was recorded.

### Reporting summary

Further information on research design is available in the Nature Portfolio Reporting Summary linked to this article.

## Supplementary information

Supplementary Information

Description of Additional Supplementary Files

Supplementary Dataset 1

Supplementary Dataset 2

Reporting Summary

## Data Availability

The experimental data used in this study are publicly available at 10.1594/PANGAEA.947116. The datasets generated by the models are available at 10.6084/m9.figshare.21441645.v1.

## Code availability

The code for the random regression forest model developed in R in this study is provided in the Supplementary Dataset 2.
