# Supplementary material for: Aerobic oxidation of methane significantly reduces global diffusive methane emissions from shallow marine waters
Source: Nat Commun. 2022 Nov 27;13:7309. doi: 10.1038/s41467-022-35082-y (PMC9701681; doi:10.1038/s41467-022-35082-y)
Supplement: Supplementary file 1 — Supplementary Information [file 41467_2022_35082_MOESM1_ESM.pdf]

Supplementary Information

Aerobic oxidation of methane significantly reduces global diffusive methane emissions from shallow marine waters

Shi-Hai Mao[1, 2, 3], Hong-Hai Zhang[1, 2, 3], Guang-Chao Zhuang[1, 2, 3, #], Xiao-Jun Li[1, 2, 3], Qiao Liu[1, 2, 3], Zhen Zhou[1, 2, 3], Wei-Lei Wang[4], Chun-Yang Li[5], Ke-Yu Lu[6], Xi-Ting Liu[7], Andrew Montgomery[8], Samantha B. Joye[9], Yu-Zhong Zhang[5, 10], Gui-Peng Yang[1, 2, 3, #]

[1]Frontiers Science Center for Deep Ocean Multispheres and Earth System, and Key Laboratory of Marine Chemistry Theory and Technology, Ministry of Education, Ocean University of China, Qingdao, 266100, China

[2]Laboratory for Marine Ecology and Environmental Science, Qingdao National Laboratory for Marine Science and Technology, Qingdao, 266237, China

[3]College of Chemistry and Chemical Engineering, Ocean University of China, Qingdao, 266100, China

[4]State Key Laboratory of Marine Environmental Science, College of Ocean and Earth Sciences, Xiamen University, Xiamen, 361102, China

[5]College of Marine Life Sciences, and Frontiers Science Center for Deep Ocean Multispheres and Earth System, Ocean University of China, Qingdao, 266100, China.

[6]Department of Earth Sciences, University College London, London WC1E 6BS, UK

[7]College of Marine Geosciences, Ocean University of China, Qingdao, 266100, China

[8]Department of Chemistry and Biochemistry, Montana State University, Bozeman, MT, 59717, USA

[9]Department of Marine Sciences, University of Georgia, Athens, GA, 30602, USA

[10]Marine Biotechnology Research Center, State Key Laboratory of Microbial Technology, Shandong University, Qingdao, 266237, China

[#]Correspondence: G.-C. Zhuang (zgc@ouc.edu.cn); G.-P. Yang (gpyang@mail.ouc.edu.cn)

Supplementary Figures

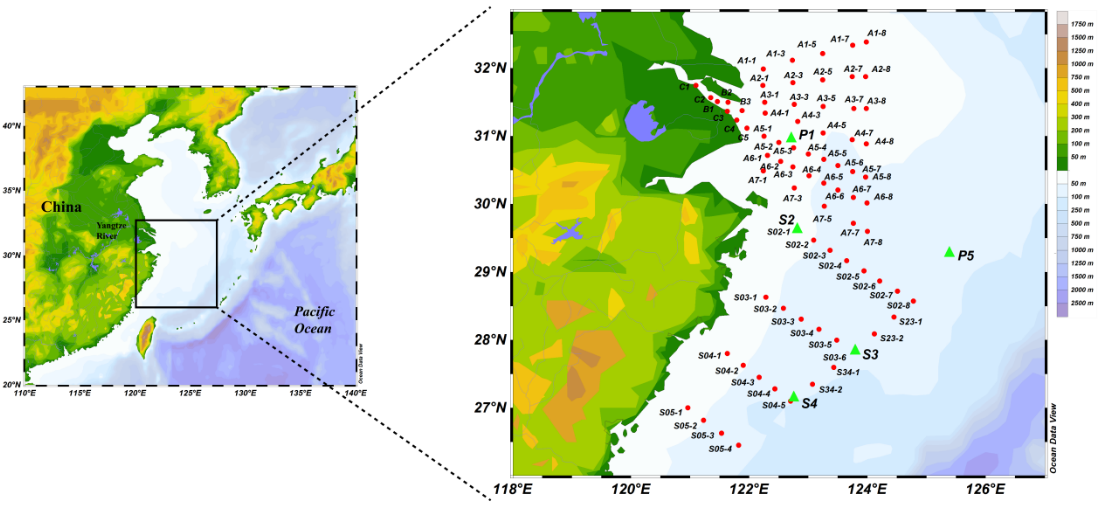

Supplementary Figure 1. Sampling sites in the East China Sea (ECS) and Yangtze River Estuary. Figure was created using Ocean Data View (version 5.5.2) [1].

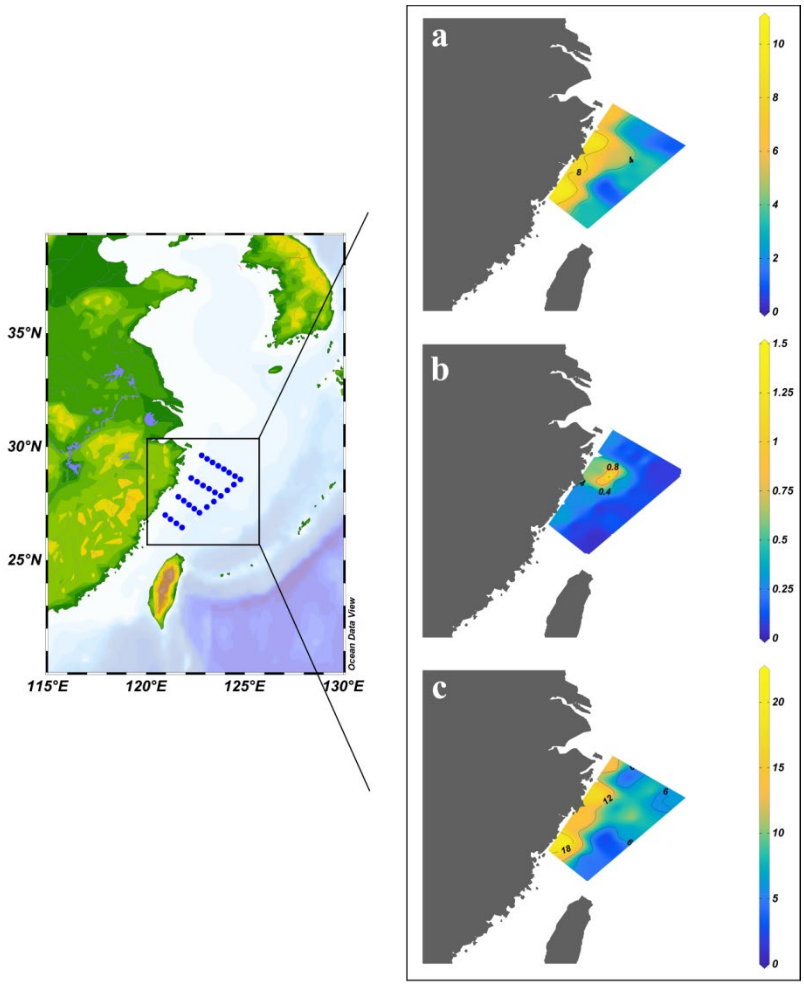

Supplementary Figure 2. **Nutrients concentrations in surface water.** Horizontal distributions of (a) dissolved inorganic nitrogen (DIN, μmol L^{-1}), (b) phosphate (PO_4^{3-}, μmol L^{-1}), and (c) silicate (SiO_3^{2-}, μmol L^{-1}) in the surface seawater of the East China sea (ECS). Figure was created with Ocean Data View (version 5.5.2)[1].

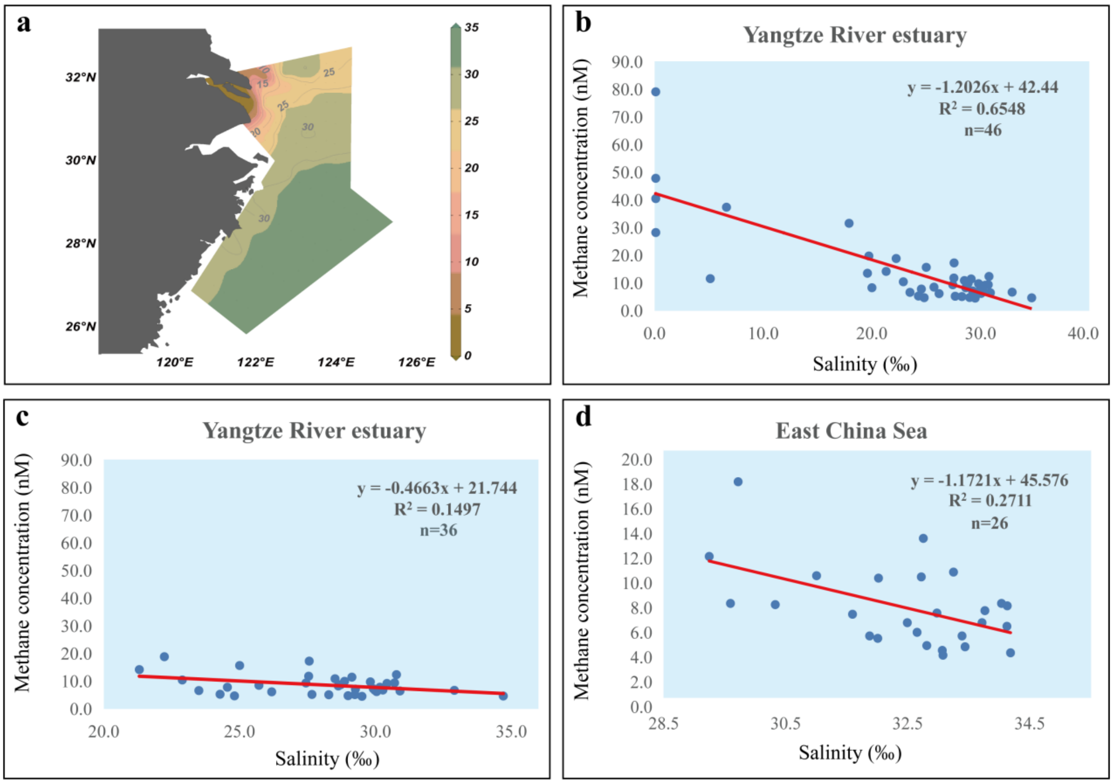

Supplementary Figure 3. **The relationships between methane concentration and salinity.** (a) Spatial distribution of salinity (‰) in the surface seawater of the Yangtze River Estuary and East China Sea. (b) The relationship between methane and salinity at all sites during the Yangzte River estuary expedition. (c) The relationship between methane and salinity > 20 during the Yangzte River estuary expedition. (d) The relationship between methane and salinity during the East China Sea expedition.

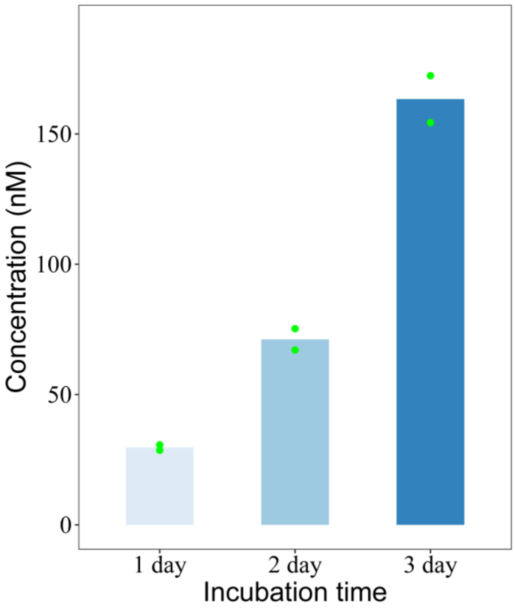

Supplementary Figure 4. **Methane emissions from sediments**. Changes of methane concentrations in the overlying seawater during incubations of sediment cores. Data are presented as the mean values of duplicate incubations.

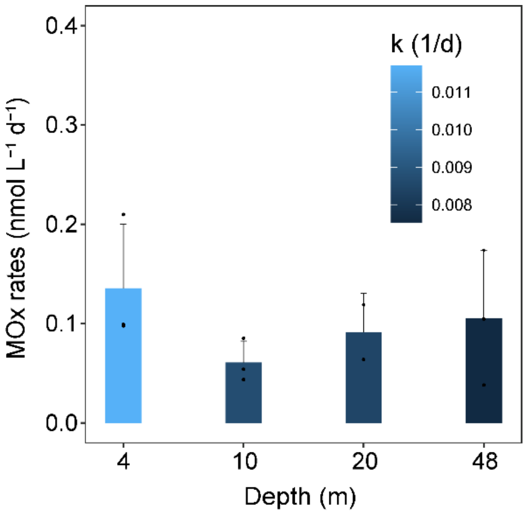

Supplementary Figure 5. **Vertical distribution of methane oxidation (MOx) rates at site S02-2 (water depth 48 m) in the East China Sea (ECS)**. The color gradient denotes the magnitude of the methane turnover rate constant (*k*, 1/d). P-value (p=0.44) is derived from one-way Analysis of Variance (ANOVA). Data are presented as mean values and error bars represent standard deviation of triplicate samples.

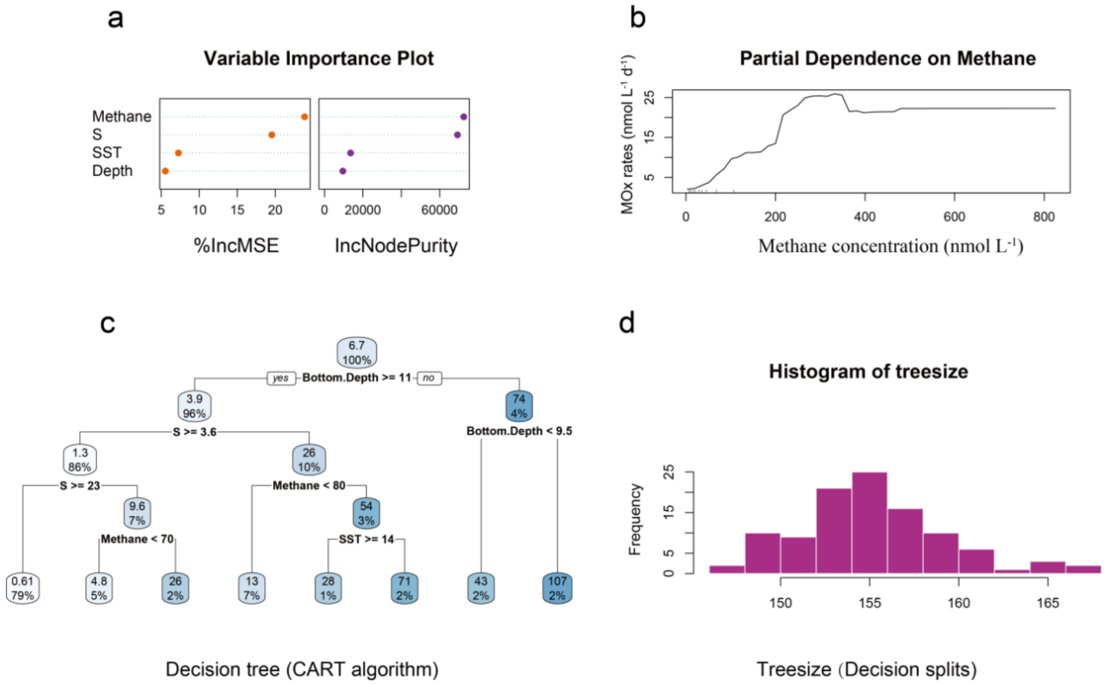

Supplementary Figure 6. **Parameters for the random regression forest (RRF) model**. (a) Assessment for the importance of predictor variables based on %IncMSE (Increase in Mean Squared Error) and IncNodePurity (Increase in Node Purity); (b) dependence between methane oxidation (MOx) rates and the main predictor variable methane concentration; (c) a single decision tree from the RRF model based on CART algorithm; (d) frequency histogram for decision splits of the RRF model.

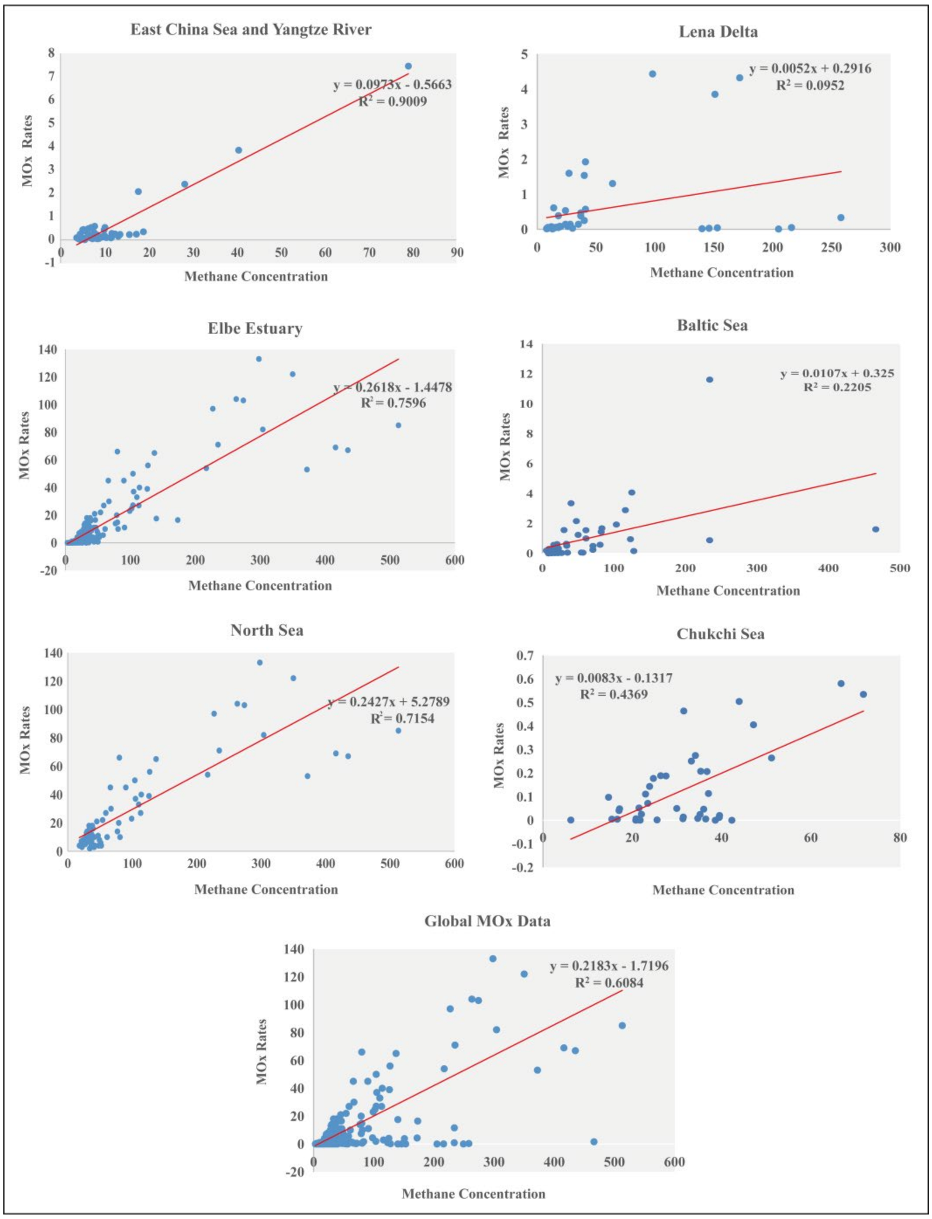

Supplementary Figure 7. Relationships between methane concentrations and methane oxidation (MOx) rates in global diffusive systems using data collected in Supplementary Table 2.

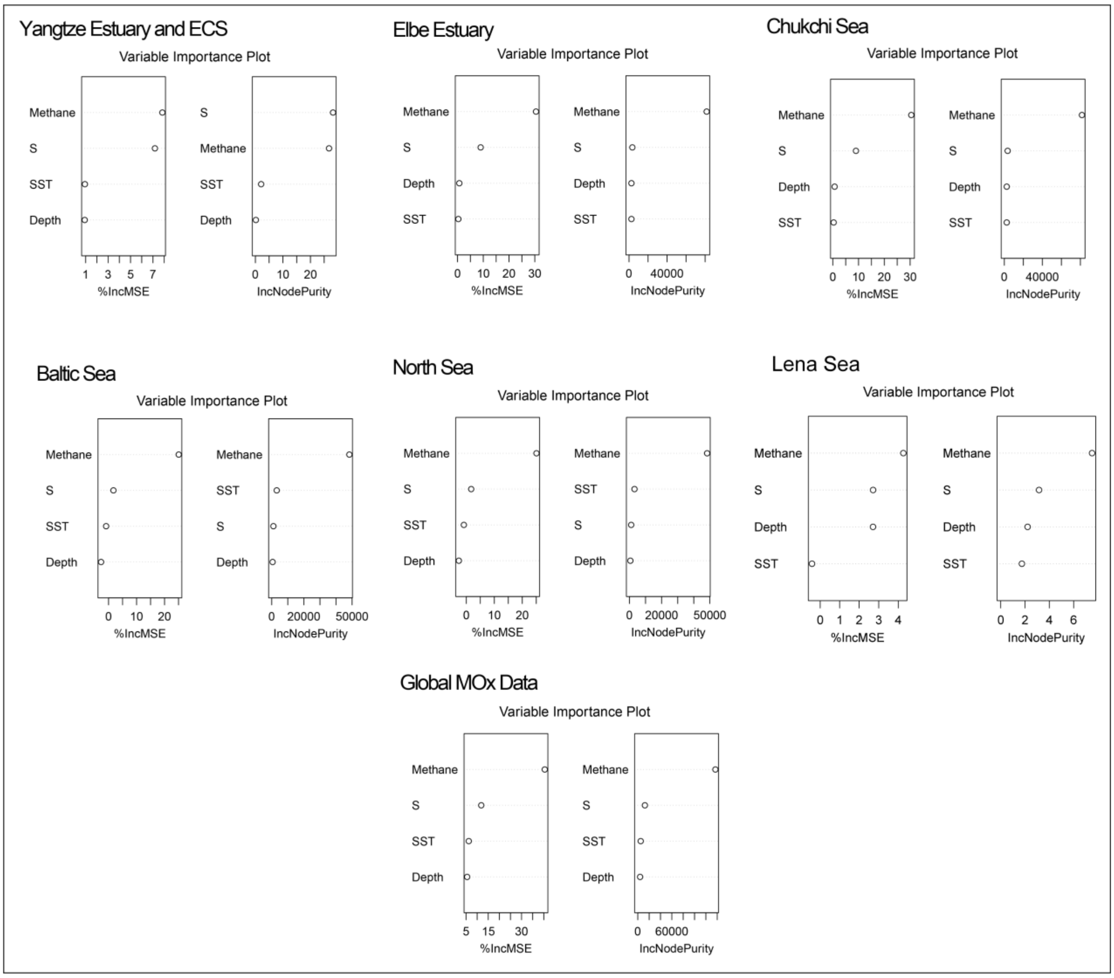

Supplementary Figure 8. The importance of predictor variables including methane, salinity (S), temperature (SST), and depth in different systems. %IncMSE: Increase in Mean Squared Error; IncNodePurity: Increase in Node Purity.

Supplementary Tables

Supplementary Table 1. Frequency of predicted functional gene families in the metagenomic sequences.

East China Sea		
Station	P6 (28.72°N, 126.14°E)	FJ2 (31.33°N, 124.5°E)
Predicted function	* Normalized abundance (***Ra***)	
Methyl coenzyme M reductase (*mcr*A)	N.D.	N.D.
Phosphoenolpyruvate phosphomutase (*pepM*)	5.41	156.8
Methylphosphonate synthase (*mpnS*)	8.83	3.17
phnC	28.51	24.14
phnD	1.73	6.25
phnE	7.28	10.90
phnI	20.06	12.55
phnJ	31.46	21.27
phnK	22.70	17.80
phnL	17.72	0.00
phnM	12.89	16.06

*Normalized ratio (***Ra***) = total reads related to function / (gene sequence length / 1000) × total number of reads / 1,000,000).

N.D.: Not detected.

Supplementary Table 2. Collection of methane oxidation (MOx) rates database for training machine learning models.

MOx Database		
Study area	***Data source***	***Data DOI***
Boknis Eck (Baltic Sea)	(Steinle et al., 2017)[2]	https://doi.org/10.1594/PANGAEA.871890
Elbe Estuary	(Bussmann et al., 2014a; Bussmann et al., 2019; Matousu et al., 2015)[3-5]	https://doi.org/10.1594/PANGAEA.833923<br>https://doi.org/10.1594/PANGAEA.897351<br>https://doi.org/10.1594/PANGAEA.855825
North Sea	(Bussmann et al., 2014b)[6]	https://doi.org/10.1594/PANGAEA.833798
Chukchi Sea	(Rogener et al., 2019)[7]	https://doi.org/10.7266/SQNEJDPR
Lena Delta	(Bussmann et al., 2016; Bussmann et al., 2020; Osudar et al., 2016)[8-10]	https://doi.org/10.1594/PANGAEA.868494<br>https://doi.org/10.1594/PANGAEA.920015<br>https://doi.org/10.1594/PANGAEA.861845
Yenisey River Estuary	(Namsaraev et al., 1995)[11]	https://doi.org/10.1594/PANGAEA.746801
East China Sea<br>Yangtze River Estuary	This study	https://issues.pangaea.de/browse/PDI-31484

Supplementary Table 3. Predicted depth profiles of methane oxidation (MOx) rates in the East China sea (ECS) using the random regression forest (RRF) model based on the previous measurements of methane concentrations and other parameters in the ECS[12].

Station	Longitude (degrees East)	Latitude (degrees North)	Bottom depth (m)	Depth (m)	Methane Concentration nmol L^{-1}	MOx Rates nmol L^{-1} d^{-1}	Depth-integrated MOx Rates µmol m^{-2} d^{-1}
L12-1	125.18	29.56	86	75	7.55	0.289	27.35
L12-1	125.18	29.56	86	30	7.99	0.370	
L12-1	125.18	29.56	86	5	8.41	0.218	
L12-2	124.86	29.13	101	96	7.74	0.484	43.11
L12-2	124.86	29.13	101	30	7.99	0.403	
L12-2	124.86	29.13	101	5	7.44	0.230	
L23-1	122.68	29.37	52	51	14.95	0.455	36.33
L23-1	122.68	29.37	52	20	23.64	0.985	
L23-1	122.68	29.37	52	5	22.09	0.662	
L23-2	122.54	29.12	51	47	16.66	0.455	16.98
L23-2	122.54	29.12	51	20	9.65	0.223	
L23-2	122.54	29.12	51	5	11.36	0.297	
L23-3	122.41	28.88	50	48	10.01	0.451	22.31
L23-3	122.41	28.88	50	20	12.54	0.411	
L23-3	122.41	28.88	50	5	17.98	0.507	
L45-1	121.46	27.59	39	36	9.93	0.259	9.71
L45-1	121.46	27.59	39	20	12.39	0.261	
L45-1	121.46	27.59	39	5	10.39	0.216	
L45-2	121.28	27.40	40	37	10.56	0.260	9.57
L45-2	121.28	27.40	40	20	9.35	0.243	
L45-2	121.28	27.40	40	5	8.21	0.211	
L45-3	121.11	27.20	41	39	5.29	0.327	9.95
L45-3	121.11	27.20	41	20	8.06	0.195	
L45-3	121.11	27.20	41	5	6.65	0.244	
S1-1	122.70	29.99	33	30	27.80	1.225	31.04
S1-1	122.70	29.99	33	20	28.46	0.990	
S1-1	122.70	29.99	33	10	13.83	0.719	
S1-1	122.70	29.99	33	5	15.65	0.635	
S1-2	123.09	30.00	50	47	13.68	0.391	22.33
S1-2	123.09	30.00	50	30	13.13	0.399	
S1-2	123.09	30.00	50	20	24.93	0.913	
S1-2	123.09	30.00	50	10	8.88	0.209	
S1-2	123.09	30.00	50	5	12.40	0.206	
S1-3	123.50	30.00	70	67	16.94	0.322	17.92
S1-3	123.50	30.00	70	50	11.06	0.257	
S1-3	123.50	30.00	70	30	11.98	0.252	
S1-3	123.50	30.00	70	20	8.56	0.241	
S1-3	123.50	30.00	70	10	8.54	0.241	
S1-3	123.50	30.00	70	5	9.87	0.200	

S1-4	124.00	30.00	61	58	10.78	0.458	18.87
S1-4	124.00	30.00	61	50	10.18	0.394	
S1-4	124.00	30.00	61	30	4.42	0.288	
S1-4	124.00	30.00	61	20	4.54	0.274	
S1-4	124.00	30.00	61	10	8.33	0.201	
S1-4	124.00	30.00	61	5	5.25	0.249	
S1-5	124.30	30.00	58	50	10.13	0.211	15.78
S1-5	124.30	30.00	58	30	9.56	0.230	
S1-5	124.30	30.00	58	20	8.84	0.265	
S1-5	124.30	30.00	58	10	10.70	0.422	
S1-5	124.30	30.00	58	5	9.30	0.345	
S1-6	124.69	30.00	60	53	18.61	0.168	15.69
S1-6	124.69	30.00	60	30	7.73	0.220	
S1-6	124.69	30.00	60	20	8.29	0.251	
S1-6	124.69	30.00	60	10	11.13	0.482	
S1-6	124.69	30.00	60	5	8.33	0.391	
S1-7	125.10	29.98	60	30	6.87	0.270	16.94
S1-7	125.10	29.98	60	20	5.98	0.244	
S1-7	125.10	29.98	60	10	4.87	0.381	
S1-7	125.10	29.98	60	5	6.23	0.246	
S1-8	125.51	30.00	64	61	8.76	0.189	17.00
S1-8	125.51	30.00	64	50	4.18	0.259	
S1-8	125.51	30.00	64	30	6.41	0.327	
S1-8	125.51	30.00	64	20	5.46	0.309	
S1-8	125.51	30.00	64	10	7.74	0.190	
S1-8	125.51	30.00	64	5	5.18	0.368	
S2-1	124.53	28.70	91	87	8.80	0.447	30.39
S2-1	124.53	28.70	91	30	8.08	0.247	
S2-1	124.53	28.70	91	5	3.70	0.217	
S2-2	124.29	28.83	78	50	10.16	0.262	22.51
S2-2	124.29	28.83	78	30	5.61	0.317	
S2-2	124.29	28.83	78	5	5.38	0.249	
S2-3	124.04	28.96	80	78	9.10	0.251	20.43
S2-3	124.04	28.96	80	30	10.30	0.249	
S2-3	124.04	28.96	80	5	4.60	0.294	
S2-4	124.79	29.09	80	76	3.51	0.286	22.71
S2-4	124.79	29.09	80	30	7.20	0.273	
S2-4	124.79	29.09	80	5	5.53	0.320	
S2-5	123.55	29.22	76	73	8.40	0.257	19.24
S2-5	123.55	29.22	76	30	9.54	0.252	
S2-5	123.55	29.22	76	5	12.46	0.249	
S2-6	123.20	29.36	71	69	14.53	0.455	29.26
S2-6	123.20	29.36	71	30	14.21	0.413	
S2-6	123.20	29.36	71	5	12.88	0.315	
S2-7	123.06	29.49	63	60	11.04	0.448	24.24
S2-7	123.06	29.49	63	20	12.51	0.363	
S2-7	123.06	29.49	63	5	9.48	0.219	
S2-8	122.81	29.62	53	50	11.01	0.448	17.73

S2-8	122.81	29.62	53	20	9.95	0.212	
S2-8	122.81	29.62	53	5	6.70	0.320	
S3-1	122.27	28.64	45	41	17.90	0.496	35.26
S3-1	122.27	28.64	45	20	14.81	0.426	
S3-1	122.27	28.64	45	10	29.28	1.784	
S3-1	122.27	28.64	45	5	18.24	0.646	
S3-2	122.52	28.51	64	61	13.50	0.431	18.55
S3-2	122.52	28.51	64	30	9.76	0.253	
S3-2	122.52	28.51	64	5	6.44	0.241	
S3-3	122.76	28.36	76	74	7.64	0.449	27.05
S3-3	122.76	28.36	76	30	6.30	0.324	
S3-3	122.76	28.36	76	5	7.64	0.213	
S3-4	123.02	28.23	81	79	9.84	0.448	29.68
S3-4	123.02	28.23	81	30	6.80	0.309	
S3-4	123.02	28.23	81	5	5.80	0.328	
S3-5	123.25	28.09	82	76	6.94	0.322	25.13
S3-5	123.25	28.09	82	30	4.61	0.290	
S3-5	123.25	28.09	82	5	5.76	0.322	
S3-6	123.51	27.96	88	76.5	14.35	0.455	30.06
S3-6	123.51	27.96	88	30	8.78	0.248	
S3-6	123.51	27.96	88	5	2.74	0.284	
S4-5	121.81	27.67	58	56	12.76	0.443	15.36
S4-5	121.81	27.67	58	30	8.95	0.241	
S4-5	121.81	27.67	58	5	7.64	0.216	
S4-6	121.63	27.78	37	33	13.75	0.259	8.85
S4-6	121.63	27.78	37	20	12.45	0.235	
S4-6	121.63	27.78	37	5	11.54	0.234	
S5-1	120.93	27.01	41	39	7.31	0.273	11.72
S5-1	120.93	27.01	41	20	6.29	0.324	
S5-1	120.93	27.01	41	5	9.61	0.211	
S5-2	121.14	26.89	58	55	5.71	0.318	18.28
S5-2	121.14	26.89	58	30	6.90	0.312	
S5-2	121.14	26.89	58	5	6.13	0.327	
S5-3	121.34	26.76	71	68	6.19	0.327	22.83
S5-3	121.34	26.76	71	30	6.59	0.320	
S5-3	121.34	26.76	71	5	5.63	0.316	
S5-4	121.55	26.64	78	76	4.80	0.290	21.90
S5-4	121.55	26.64	78	30	4.38	0.275	
S5-4	121.55	26.64	78	5	2.86	0.276	
S5-5	121.75	26.52	90	75	4.96	0.310	28.35
S5-5	121.75	26.52	90	30	6.64	0.319	
S5-5	121.75	26.52	90	5	6.39	0.322	
S5-6	121.96	26.39	101	100	5.29	0.316	28.76
S5-6	121.96	26.39	101	30	8.06	0.247	
S5-6	121.96	26.39	101	5	2.69	0.276	
Average							22.08

Supplementary Table 4. Sites information for methane production incubation experiments

Station	Longitude (°E)	Latitude (°N)	Depth (m)	Salinity (‰)	Phosphate (μM)	Nitrate (μM)	Nitrite (μM)	Ammonium (μM)
P1	122.72	30.96	19.0	30.61	0.36	17.20	0.11	N.M.
P5	125.39	29.27	87.0	34.19	0.33	3.98	0.03	N.M.
S4	122.75	27.15	104	34.51	0.22	2.46	0.25	N.M.
S2	122.81	29.62	47.9	29.94	0.34	0.83	0.49	3.85
S3	123.78	27.84	87.5	34.27	0.32	0.15	0.55	N.M.

N.M.: Not measured.

Supplementary Table 5. Different treatments for incubation experiments.

Incubation	Sampling station	Group	Substrate（μM）								
			C	N	Pi	MPn	DMSP	TMA	Methanol	Acetate	MeSH
Incubation	P1、P5、S4、	MPn				1					
		DMSP					1				
		TMA						1			
		Methanol							1		
		Acetate								1	
		[b]MeSH									1
		Control									
	S2	Control									
		[#]C+N	1060	160							
		[#]C+N+MPn	1060	160		10					
	S3	Control									
		MPn				5					
	P5	Control									
		[#]C+N	1060	160							
		[#]C+N+MPn	1060	160		10					
		[#]C+N+Pi	1060	160	10						
		[#]C+N+MPn+[a]CH_3	1060	160		10					

[a] CH_3F (Methyl Fluoride, China Institute of Metrology) was added in the headspace (1 kPa) as the methane oxidation inhibitor.

[b] MeSH taken from the Standard Gas Generator (Model 500, VICI, USA) was added in the headspace at a final concentration of 1 μM.

[#] C+N / C:N represents the correction of carbon and nitrogen in incubated seawater using glucose (Sigma) and nitrate (Sinopharm Chemical Reagent Company, Shanghai) at a ratio of 106:16, respectively.

Supplementary References

1. Schlitzer, R. Ocean Data View. http://odv.awi.de (2012).
2. Steinle, L. et al. Physico-chemical data including methane concentrations, as well as methane oxidation rates, measured at time-series station Boknis Eck (Baltic Sea) from 2012-2014. PANGAEA https://doi.org/10.5194/bg-14-1631-2017 (2017).
3. Bussmann, I., Osudar, R. & Matousu, A. Methane concentrations and methane oxidation rates from Oct 2010 - March 2012 in the Elbe Estuary, from Hamburg to Cuxhaven, Germany. PANGAEA https://doi.org/10.1594/PANGAEA.833923 (2014a).
4. Bussmann, I., Hackbusch, S. & Warnstedt, J. Methane concentrations and methane oxidation rates from Jan 2013 - Nov 2014 in the Elbe Estuary, from Hamburg to Helgoland, Germany. PANGAEA https://doi.org/10.1594/PANGAEA.897351 (2019).
5. Matousu, A., Osudar, R., Simek, K. & Bussmann, I. Methane concentrations and methane oxidation rates from June 2012 - June 2013 in the Elbe Estuary, from Hamburg to Cuxhaven, Germany. PANGAEA https://doi.org/10.1594/PANGAEA.855825 (2015).
6. Bussmann, I., Osudar, R. & Matousu, A. Methane concentrations and methane oxidation rates from Oct 2010 - Jun 2012 on a transect from Cuxhaven to Helgoland, North Sea, Germany. PANGAEA https://doi.org/10.1594/PANGAEA.833798 (2014b).
7. Rogener, M. K. et al. Methane concentration and oxidation rates in water column data collected aboard the R/V Sikuliaq in the northern Chukchi Sea from 2017-08-11 to 2017-08-20. Gulf of Mexico Research Initiative Information and Data Cooperative (GRIIDC) https://doi.org/10.7266/SQNEJDPR (2019)
8. Bussmann, I., Hackbusch, S., Schaal, P. & Wichels, A. Methane concentration and oxidation in the Lena Delta, September 2013. PANGAEA https://doi.org/10.1594/PANGAEA.868494 (2016).
9. Bussmann, I., Fedorova, I. V., Juhls, B., Overduin, P. P. & Winkel, M. Dissolved methane concentrations and oxidation rates in the Lena Delta area, 2016-2018. PANGAEA https://doi.org/10.1594/PANGAEA.920015 (2020).
10. Osudar, R. et al. (Table 1) Methane concentrations, MOX rates and MOB abundance in arctic aquatic ecosystems of the Lena Delta, Northeast Siberia. PANGAEA https://doi.org/10.1594/PANGAEA.861845 (2016).
11. Namsaraev, B. B. et al. Bacterial methane oxidation rates in waters and sediments of the Kara Sea and the Yenisey River estuary. PANGAEA https://doi.org/10.1594/PANGAEA.746801 (1995).
12. Zhai, X., Li, J. L., Zhang, H., Tan, D. D. & Yang, G. Spatial distribution and biogeochemical cycling of dimethylated sulfur compounds and methane in the East China Sea during spring. *J. Geophys. Res. - Oceans* **124,** (2018).
