# Supplementary material for: Aerobic oxidation of methane significantly reduces global diffusive methane emissions from shallow marine waters
Source: Nat Commun. 2022 Nov 27;13:7309. doi: 10.1038/s41467-022-35082-y (PMC9701681; doi:10.1038/s41467-022-35082-y)
Supplement: Supplementary file 2 — Description of Additional Supplementary Files [file 41467_2022_35082_MOESM2_ESM.pdf]

Description of Additional Supplementary Files:

Supplementary Dataset 1: Information of the functionally ratified protein sequences obtained from the National Center for Biotechnology Information (NCBI) database (https://www.ncbi.nlm.nih.gov/).

Supplementary Dataset 2: The code for the random regression forest model.
