# Supplementary material for: Aerobic oxidation of methane significantly reduces global diffusive methane emissions from shallow marine waters
Source: Nat Commun. 2022 Nov 27;13:7309. doi: 10.1038/s41467-022-35082-y (PMC9701681; doi:10.1038/s41467-022-35082-y)
Supplement: Supplementary file 4 — Supplementary Dataset 2 [file 41467_2022_35082_MOESM4_ESM.pdf]

Supplementary Dataset 2

Aerobic oxidation of methane significantly reduces global diffusive methane emissions from shallow marine waters

Shi-Hai Mao[1, 2, 3], Hong-Hai Zhang[1, 2, 3], Guang-Chao Zhuang[1, 2, 3, #], Xiao-Jun Li[1, 2, 3], Qiao Liu[1, 2, 3], Zhen Zhou[1, 2, 3], Wei-Lei Wang[4], Chun-Yang Li[5], Ke-Yu Lu[6], Xi-Ting Liu[7], Andrew Montgomery[8], Samantha B. Joye[9], Yu-Zhong Zhang[5, 10], Gui-Peng Yang[1, 2, 3, #]

[1]Frontiers Science Center for Deep Ocean Multispheres and Earth System, and Key Laboratory of Marine Chemistry Theory and Technology, Ministry of Education, Ocean University of China, Qingdao, 266100, China

[2]Laboratory for Marine Ecology and Environmental Science, Qingdao National Laboratory for Marine Science and Technology, Qingdao, 266237, China

[3]College of Chemistry and Chemical Engineering, Ocean University of China, Qingdao, 266100, China

[4]State Key Laboratory of Marine Environmental Science, College of Ocean and Earth Sciences, Xiamen University, Xiamen, 361102, China

[5]College of Marine Life Sciences, and Frontiers Science Center for Deep Ocean Multispheres and Earth System, Ocean University of China, Qingdao, 266100, China.

[6]Department of Earth Sciences, University College London, London WC1E 6BS, UK

[7]College of Marine Geosciences, Ocean University of China, Qingdao, 266100, China

[8]Department of Chemistry and Biochemistry, Montana State University, Bozeman, MT, 59717, USA

[9]Department of Marine Sciences, University of Georgia, Athens, GA, 30602, USA

[10]Marine Biotechnology Research Center, State Key Laboratory of Microbial Technology, Shandong University, Qingdao, 266237, China

[#]Correspondence: G.-C. Zhuang (zgc@ouc.edu.cn); G.-P. Yang (gpyang@mail.ouc.edu.cn)

Source Code

Code for developing random regression forest prediction models.

```
#Loading R packages
library (randomForest)
library(tidyverse)
library (skimr)
library (DataExplorer)
library(lattice)
library(ggplot2)
library (caret)
#Data loading
MOX_DAT <- read.table (file="MOX_DAT.csv", sep=",", header=T)
MOX_PRED_DAT <- read.table (file="MOX_PRED_DAT.csv", sep=",", header=T)
skim(MOX_DAT)
plot_missing(MOX_DAT)
hist (MOX_DAT$MOx, breaks = 50)
set.seed (821)
trains <- createDataPartition (y = MOX_DAT$MOx, p = 0.75, list = F)
traindata <- MOX_DAT [trains, ]
testdata <- MOX_DAT [-trains, ]
colnames (MOX_DAT)
FORM_REG_GS <- as.formula (paste0 ("MOx~", paste (colnames (traindata) [1:4], collapse = " +
") ) )
#build random regression forest prediction models
set.seed (6162)
RRF_MOD <- randomForest (FORM_REG_GS, data = traindata, ntree=105, mtry = 4, importance
= T)
RRF_MOD
plot (RRF_MOD, main = "ERROR & TREES")
```

```
#assessment for the importance of predictor variables
varImpPlot (RRF_MOD, main = "Variable Importance Plot")
#dependence between MOx rates and methane concentration
partialPlot (x = RRF_MOD, pred.data = traindata, x.var = Methane)
plot (MOx ~ Methane, data = traindata)
#training results
trainpred <-predict(RRF_MOD, newdata = traindata)
defaultSummary (data.frame (obs = traindata$MOx, pred = trainpred))
plot (x = traindata$MOx, y = trainpred, xlab = "Observation data", ylab = "Prediction data")
abline (a = 0, b= 1, col = "red", lwd = 2.5, lty = "dashed")
#testing results
testpred <-predict (RRF_MOD, newdata = testdata)
defaultSummary (data.frame (obs = testdata$MOx, pred = testpred) )
plot (x =testdata$MOx, y = testpred, xlab = "Observation data", ylab = "Prediction data")
abline (a =0, b=1, col = "red" , lwd = 2.5, lty = "dashed")
#Centralized presentation of training and testing results
PRED_RESULT = data.frame (OBS =c (traindata$MOx, testdata$MOx), PRED = c (trainpred,
testpred), group =c(rep ("Train", length (trainpred)), rep ("Test", length (testpred) ) ) )
ggplot (PRED_RESULT, aes (x = OBS , y = PRED, fill = group, colour = group)) + geom_point
(size=3) + geom_smooth (method = "lm", se=F, size = 1.0) + scale_color_brewer (palette = "Set1")
#model prediction
MOx_Pred <-predict (RRF_MOD, newdata = MOX_PRED_DAT)
write.csv(MOx_Pred, "MOx_Pred.csv")
```
