# Supplementary material for: Aerobic oxidation of methane significantly reduces global diffusive methane emissions from shallow marine waters
Source: Nat Commun. 2022 Nov 27;13:7309. doi: 10.1038/s41467-022-35082-y (PMC9701681; doi:10.1038/s41467-022-35082-y)
Supplement: Supplementary file 5 — Reporting Summary [file 41467_2022_35082_MOESM5_ESM.pdf]

nature portfolio

Corresponding author(s): Guang-Chao Zhuang

Last updated by author(s): Nov 4, 2022

Reporting Summary

Nature Portfolio wishes to improve the reproducibility of the work that we publish. This form provides structure for consistency and transparency in reporting. For further information on Nature Portfolio policies, see our Editorial Policies and the Editorial Policy Checklist.

Statistics

For all statistical analyses, confirm that the following items are present in the figure legend, table legend, main text, or Methods section.

n/a	Confirmed	
☐	☒	The exact sample size (*n*) for each experimental group/condition, given as a discrete number and unit of measurement
☐	☒	A statement on whether measurements were taken from distinct samples or whether the same sample was measured repeatedly
☐	☒	The statistical test(s) used AND whether they are one- or two-sided<br>*Only common tests should be described solely by name; describe more complex techniques in the Methods section.*
☒	☐	A description of all covariates tested
☒	☐	A description of any assumptions or corrections, such as tests of normality and adjustment for multiple comparisons
☐	☒	A full description of the statistical parameters including central tendency (e.g. means) or other basic estimates (e.g. regression coefficient) AND variation (e.g. standard deviation) or associated estimates of uncertainty (e.g. confidence intervals)
☒	☐	For null hypothesis testing, the test statistic (e.g. *F*, *t*, *r*) with confidence intervals, effect sizes, degrees of freedom and *P* value noted<br>*Give P values as exact values whenever suitable.*
☒	☐	For Bayesian analysis, information on the choice of priors and Markov chain Monte Carlo settings
☒	☐	For hierarchical and complex designs, identification of the appropriate level for tests and full reporting of outcomes
☒	☐	Estimates of effect sizes (e.g. Cohen's *d*, Pearson's *r*), indicating how they were calculated

Our web collection on statistics for biologists contains articles on many of the points above.

Software and code

Policy information about availability of computer code

Data collection	Geochemical analyses were conducted with Agilent GC-8890 gas chromatography, nutrient anaylzer (SEAL AA3), Ticarb 3110TR scintillation counter.
Data analysis	Geochemical data were mapped using Ocean Date View v.5.5.2.<br>Data for incubation experiments were analyzed using R v.4.0.3.<br>Global methane oxidation rates were predicted with random regression forest machine-learning models using R v.4.0.3.

For manuscripts utilizing custom algorithms or software that are central to the research but not yet described in published literature, software must be made available to editors and reviewers. We strongly encourage code deposition in a community repository (e.g. GitHub). See the Nature Portfolio guidelines for submitting code & software for further information.

Data

Policy information about availability of data

All manuscripts must include a data availability statement. This statement should provide the following information, where applicable:

- Accession codes, unique identifiers, or web links for publicly available datasets
- A description of any restrictions on data availability
- For clinical datasets or third party data, please ensure that the statement adheres to our policy

The data used in this study are publicly available at https://doi.org/10.1594/PANGAEA.947116. The datasets generated by the models are available at https://

doi.org/10.6084/m9.figshare.21441645.v1.

Human research participants

Policy information about studies involving human research participants and Sex and Gender in Research.

Reporting on sex and gender	No human research was conducted in this study.
Population characteristics	See above.
Recruitment	See above.
Ethics oversight	See above.

Note that full information on the approval of the study protocol must also be provided in the manuscript.

Field-specific reporting

Please select the one below that is the best fit for your research. If you are not sure, read the appropriate sections before making your selection.

☐ Life sciences ☐ Behavioural & social sciences ☒ Ecological, evolutionary & environmental sciences

For a reference copy of the document with all sections, see nature.com/documents/nr-reporting-summary-flat.pdf

Ecological, evolutionary & environmental sciences study design

All studies must disclose on these points even when the disclosure is negative.

Study description	This study was conducted to investigate methane sources and fate in shallow waters using a variety of biogeochemical techniques and mapped global rates of aerobic oxidation of methane (MOx) in shallow well-mixed waters by training machine-learning models. We chose n = 3 for MOx rate measurements. A total of 427 datapoints for MOx rates and 6633 datapoints for methane concentrations collected from this study and previous database were used to generate predictions of global MOx rates in shallow waters using random regression forest (RRF) machine-learning method.
Research sample	Seawater samples were collected from the Yangtze River estuary and the East China Sea (ECS). The hydrological and biogeochemical features make Yangtze estuary and ECS an appropriate model for estuary-shelf continuum systems across the globe.
Sampling strategy	Samples were collected from a total of 76 sites using a Seabird 911 CTD-Niskin rosette system, and triplicate measurements for rates experiments were conducted to give a robust statistical analysis.
Data collection	Shi-Hai Mao, Xiao-Jun Li, Qiao Liu and Zhen Zhou collected samples and conducted geochemical and microbial activity rate assays; Shi-Hai Mao, Wei-Lei Wang, and Guang-Chao Zhuang developed machine-learning models; Chun-Yang Li and Yu-Zhong Zhang conducted the metagenomic analyses.
Timing and spatial scale	This study included a variety of biogeochemical analyses spanning from 2019.12.26 to 2022.12.18, including laboratory experiments, field work during expeditions and post-cruise analyses of samples.
Data exclusions	No data were excluded.
Reproducibility	All experiments were conducted with good reproducibility. All attempts to repeat the experiments were successful.
Randomization	Not relevant as same researchers took responsibility for individual analysis.
Blinding	Blinding is not relevant as all data analyses were performed by the same groups of researchers from the authors.

Did the study involve field work? ☒ Yes ☐ No

Field work, collection and transport

Field conditions	Seawater samples were collected from the Yangtze River Estuary and East China Sea onboard the R/Vs "Runjiang 1", "Zheyuke 2" and "Xiangyanghong 18" during expeditions in March-April and July 2021.
Location	Yangtze River Estuary and East China Sea (~26°N-33°N, ~121°E-124°E, 7m-104m).
Access & import/export	Seawater samples were collected onboard the R/Vs "Runjiang 1", "Zheyuke 2" and "Xiangyanghong 18" during scientific expeditions,

	which were funded by Shiptime Sharing Project of National Natural Science Foundation of China.
Disturbance	Disturbance on coastal environments was minimized during sampling.

Reporting for specific materials, systems and methods

We require information from authors about some types of materials, experimental systems and methods used in many studies. Here, indicate whether each material, system or method listed is relevant to your study. If you are not sure if a list item applies to your research, read the appropriate section before selecting a response.

Materials & experimental systems

n/a	Involved in the study	
☒	☐	Antibodies
☒	☐	Eukaryotic cell lines
☒	☐	Palaeontology and archaeology
☒	☐	Animals and other organisms
☒	☐	Clinical data
☒	☐	Dual use research of concern

Methods

n/a	Involved in the study	
☒	☐	ChIP-seq
☒	☐	Flow cytometry
☒	☐	MRI-based neuroimaging
